# Differential effect with septal and apical RV pacing on ventricular activation in patients with left bundle branch block assessed by non-invasive electrical imaging and *in silico* modelling

**DOI:** 10.1007/s10840-019-00567-2

**Published:** 2019-06-14

**Authors:** T. Jackson, S. Claridge, J. Behar, B. Sieniewicz, J. Gould, B. Porter, B. Sidhu, C. Yao, A. Lee, S. Niederer, C. A. Rinaldi

**Affiliations:** 1 Department of Imaging Sciences, St Thomas’ Hospital, King’s College London, London, SE1 UK; 2 Department of Cardiology, Salisbury NHS Foundation Trust, Salisbury, Wiltshire SP2 8BJ UK; 3 Medtronic/CardioInsight, Cleveland, OH USA; 4 Guy’s and St Thomas’ NHS Trust, King’s College London, London, SE1 9RT UK

**Keywords:** Cardiac resynchronization therapy, Left bundle branch block, RV septal pacing, Body surface mapping, Computer modelling

## Abstract

**Purpose:**

It is uncertain whether right ventricular (RV) lead position in cardiac resynchronization therapy impacts response. There has been little detailed analysis of the activation patterns in RV septal pacing (RVSP), especially in the CRT population. We compare left bundle branch block (LBBB) activation patterns with RV pacing (RVP) within the same patients with further comparison between RV apical pacing (RVAP) and RVSP.

**Methods:**

Body surface mapping was undertaken in 14 LBBB patients after CRT implantation. Nine patients had RVAP, 5 patients had RVSP. Activation parameters included left ventricular total activation time (LVtat), biventricular total activation time (VVtat), interventricular electrical synchronicity (VVsync), and dispersion of left ventricular activation times (LVdisp). The direction of activation wave front was also compared in each patient (wave front angle (WFA)). *In silico* computer modelling was applied to assess the effect of RVAP and RVSP in order to validate the clinical results.

**Results:**

Patients were aged 64.6 ± 12.2 years, 12 were male, 8 were ischemic. Baseline QRS durations were 157 ± 18 ms. There was no difference in VVtat between RVP and LBBB but a longer LVtat in RVP (102.8 ± 19.6 vs. 87.4 ± 21.1 ms, *p* = 0.046). VVsync was significantly greater in LBBB (45.1 ± 20.2 vs. 35.9 ± 17.1 ms, *p* = 0.01) but LVdisp was greater in RVP (33.4 ± 5.9 vs. 27.6 ± 6.9 ms, *p* = 0.025). WFA did rotate clockwise with RVP vs. LBBB (82.5 ± 25.2 vs. 62.1 ± 31.7 ^o^*p* = 0.026). None of the measurements were different to LBBB with RVSP; however, the differences were preserved with RVAP for VVsync, LVdisp, and WFA. *In silico* modelling corroborated these results.

**Conclusions:**

RVAP activation differs from LBBB where RVSP appears similar.

**Trial registration:**

(ClinicalTrials.gov identifier: NCT01831518)

**Electronic supplementary material:**

The online version of this article (10.1007/s10840-019-00567-2) contains supplementary material, which is available to authorized users.

## Introduction

Patients with left bundle branch block (LBBB) are more likely to benefit from cardiac resynchronization therapy (CRT) than other intrinsic conduction delays establishing this activation pattern as the primary target for response to resynchronization [1–3]. Standard delivery of CRT with biventricular pacing incorporates a right ventricular (RV) and left ventricular (LV) lead. Positioning of the LV lead to areas of delayed electrical activation is felt to be important to achieve response [4]; however, the long-term impact, if any, of the RV lead position in CRT is less clear. The SEPTAL CRT study demonstrated that septal RV pacing in CRT was non-inferior to apical RV pacing for LV reverse remodelling at 6 months with no difference in the clinical outcome [5]. Kutiyfa et al. similarly found no benefit with non-apical pacing and a suggestion of increased arrhythmic events [6], and a recent meta-analysis reported that the effects of RV apical and septal pacing on LV remodelling and functional status were similar [7].

RV apical pacing has been used as a surrogate for LBBB [8]; however, previous detailed analysis has demonstrated differences between activation patterns between right ventricular pacing (RVP) and LBBB with reduction in transeptal activation time, prolonged RV activation, more apical to basal activation with RVP, and shifting of the latest activated region [9, 10]. Activation patterns in RV septal pacing (RVSP) have had little detailed analysis, especially within the CRT population. Knowledge of the pattern of LV activation according to RV pacing site may be important especially as positioning of the LV lead is often guided by electrical measurements (Q-LV) [11] and the site of latest LV activation may differ depending on the RV stimulation site.

We hypothesised that RVSP would produce a different activation pattern to RV apical pacing (RVAP) that would be more likely to mimic LBBB and therefore alter the latest activated LV segment, changing the target segment for LV lead placement. To study this, we evaluated ventricular activation patterns with non-invasive electroanatomical body surface mapping in patients with LBBB receiving CRT during intrinsic activation and RV pacing (including apical and septal pacing). We also performed *in silico* modelling to validate the activation patterns observed with septal and apical RV pacing.

## Methods

### Inclusion criteria

The local ethics authority approved the study and all patients provided written informed consent; the study complies with the Declaration of Helsinki. Consecutive patients with an indication for CRT (NYHA II-IV heart failure, ejection fraction ≤ 35%, QRS duration ≥ 120 ms) and LBBB were included in the study.

### Body surface mapping

Biventricular pacing was undertaken using standard techniques. Positions of the RV leads were not predetermined but at the operators’ discretion and dependent upon optimal electrical parameters. LV lead position was targeted at posterolateral placement within the constraints of the patients’ coronary sinus anatomy in all patients. RV pacing position was confirmed on biplane chest radiograph post-procedure. Biventricular pacing was not programmed directly after implant as body surface mapping was undertaken the following day or at the earliest opportunity, and the device was turned on following the body surface mapping protocol.

Non-invasive ventricular epicardial activation maps were acquired using 252 electrode high-resolution ECG mapping system (ecVUE, CardioInsight Technologies Inc., Cleveland, OH). Body surface potentials were collected from the 252 electrodes positioned around the thorax supported in a single-use vest; following this, a thoracic computer tomography (CT) scan was performed in order to orientate each electrode to the epicardial shell. Subsequent to segmentation of the cardiac silhouette and the electrode positions from the thoracic CT images, 1500 unipolar electrograms were reconstructed as previously described [12].

### Data analysis

**Fig. 1:**
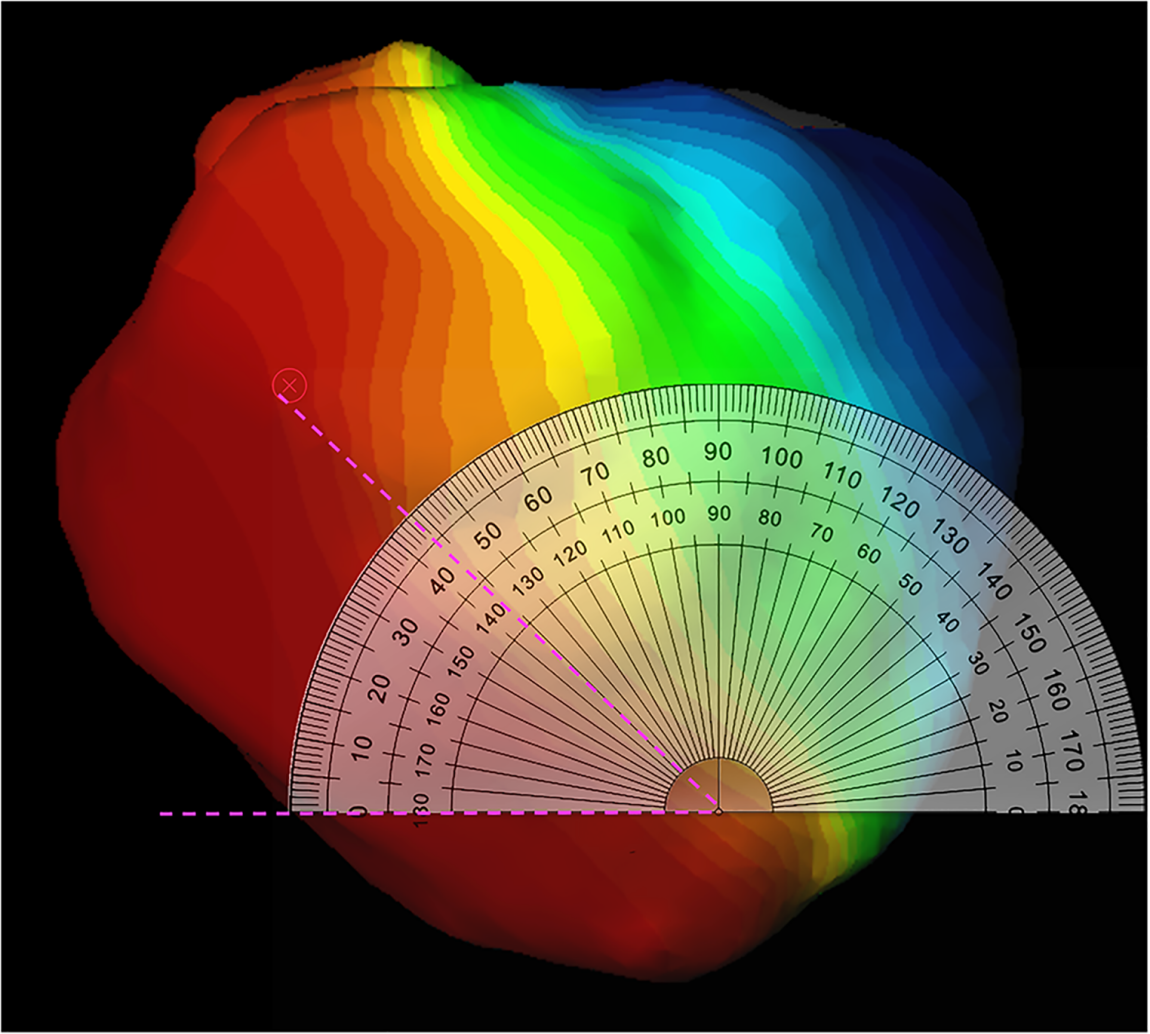
Screenshot of calculation of wave front angle. On-screen protractor (Protractor Version 10.0, Softlibs) superimposed on the activation map. The predominant wave front angle is then measured (pink dashed lines); the angle of the same isochrones is then directly compared between the right ventricular pacing map and the LBBB map as both activation maps are identically aligned

The raw data was analysed with ecSYNC software (CardioInsight Technologies Inc., Cleveland, OH) in order to derive 4 key activation metrics: VVtat (global biventricular total activation time); VVsync (global left/right ventricular electrical synchrony); LVtat (left ventricular total activation time); LVdisp (global left ventricular dispersion of activation) (see supplementary material for further information). The activation wave front angle was assessed from a screenshot of the isochrone maps for intrinsic and RVP with the cardiac shell orientated in a left anterior oblique cranial view where the basal to apical distance was maximal. The wave front angle was measured using an on-screen protractor application over each activation map screenshot (Protractor version 10.0, Softlibs) (Fig. 1). The angle measured was a user-selected most prominent isochrone line on each individual; as the cardiac geometry for the intrinsic and RVP activation maps are identically aligned, each patient’s LBBB map generates a reference wave front angle for RV pacing; the same isochrone was selected when comparing between intrinsic and RVP. The investigator was blinded for which patient and whether paced or intrinsic for each pair of maps. In order to define the final American Heart Association segments (16 segment model) to be activated, these final segments had to contain the latest isochrone on the activation map; in total, 30 isochrones were present on each map. For each patient on the intrinsic and the RVP map, there can be more than one segment which contains the final isochrone.

### *In silico* modelling

We performed *in silico* computer modelling [13] to assess the effect of RVAP and RVSP on endocardial and epicardial activation. A patient-specific mesh of a heart failure patient who underwent CRT was created from segmented MR images, as described previously [13]. Intrinsic LBBB was modelled by activating the RV at the anterior endocardial free wall [14], with a fast endocardium layer in the RV [15]. Simulations of RVAP at the anterior, posterior, and mid regions of the RV apex and RVSP were also modelled. The spread of the electrical activation across the ventricles for the 5 different scenarios (LBBB, 3 RVAP, RVSP) was simulated using the Cardiac Arrhythmia Research Package (CARP) [16].

### Statistical analysis

Statistical analysis was performed on PASW Statistics 21 (SPSS Inc., Chicago, IL, USA). The Kolmogorov-Smirnov one-sample test was used to ensure variables were normally distributed. Continuous variables were expressed as mean ± SD. Group comparisons were performed using an independent-samples *t* test for normally distributed data, and the Mann-Whitney *U* test if deemed non-parametric. Values of *p* < 0.05 were considered statistically significant.

## Results

### Body surface mapping

Fourteen patients were studied. Mean age was 64.4 ± 12.1 years, 12 were male, 8 had an ischemic aetiology. The mean QRS duration was 159.9 ± 20.8 ms and all had LBBB. Nine patients (64%) were implanted with a RV apical lead and 5 (36%) were implanted with a mid septal RV lead. The devices implanted were St. Jude Medical Quadra Assura (8 patients), St. Jude Medical Quadra Allure (2 patients), Boston Visionist, Medtronic Viva Quad, Medtronic Brava Quad, and Sorin Paradym (each in 1 patient). There was no difference in baseline demographics between the two groups (Table 1).

**Table 1:** Baseline demographics of all patients and separated into those with an apical or septal right ventricular lead. Values are mean ± standard deviation or absolute value (%)

	All patients (*n* = 14)	Apical (*n* = 9)	Septal (*n* = 5)	*p* value
Age	64.4 ± 12.1	63.3 ± 9.6	66.4 ± 17.0	0.67
Female	2 (14%)	1 (11%)	1 (20%)	1.0
Ischemic aetiology	8 (57%)	5 (56%)	3 (60%)	1.0
QRS duration (ms)	159.9 ± 20.8	153.2 ± 20.5	172.0 ± 16.9	0.11
Ejection fraction (%)	26.6 ± 5.3	26.0 ± 4.3	27.8 ± 7.3	0.16

**Fig. 2:**
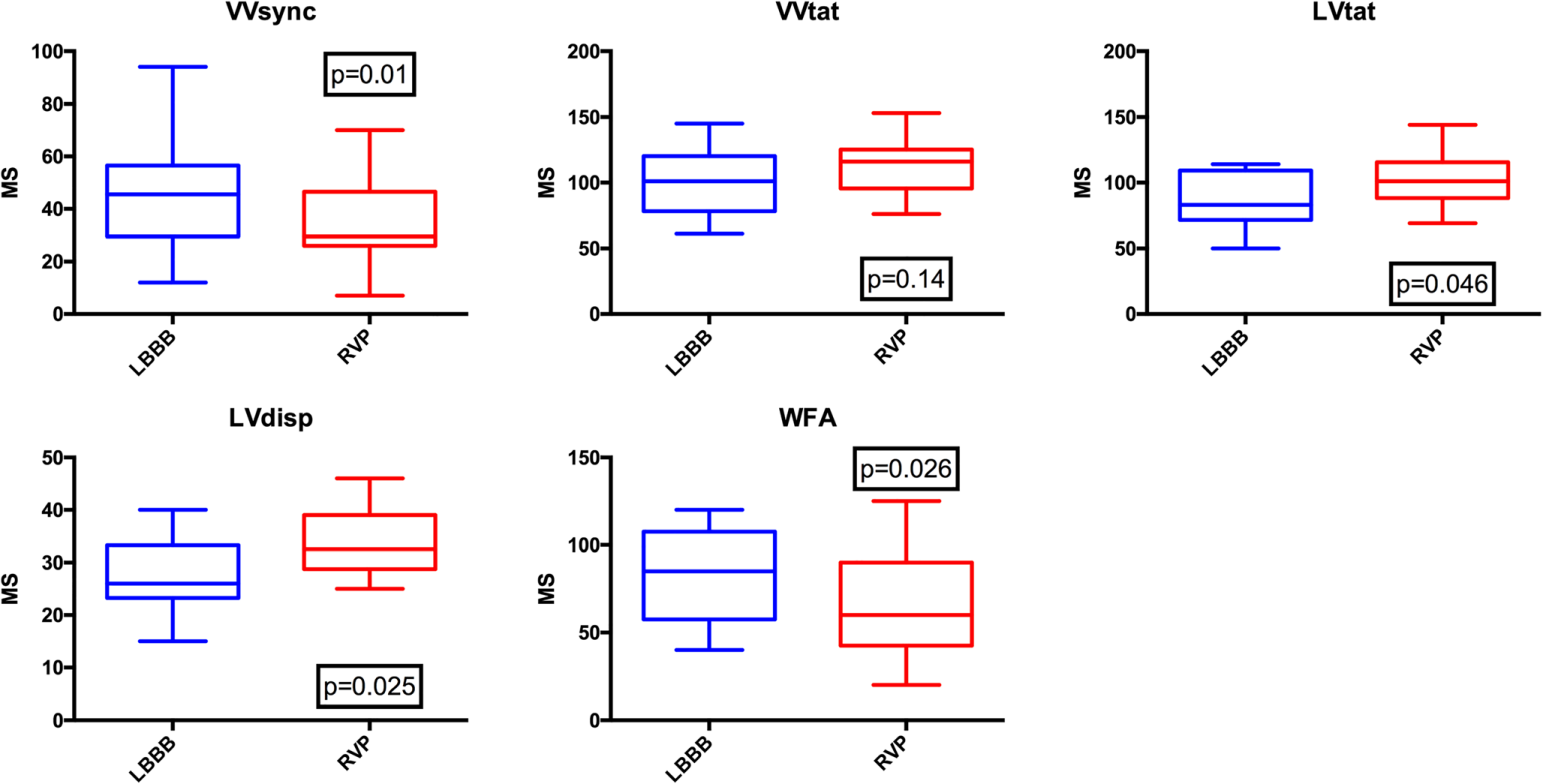
Box and whisker plots for activation times and wave front angles for intrinsic left bundle branch block (LBBB) and right ventricular pacing (RVP). VVsync—right/left ventricular electrical synchrony, VVtat—biventricular total activation time, LVtat—left ventricular total activation time, LVdisp—left ventricular dispersion of activation, WFA—wave front angle

**Fig. 3:**
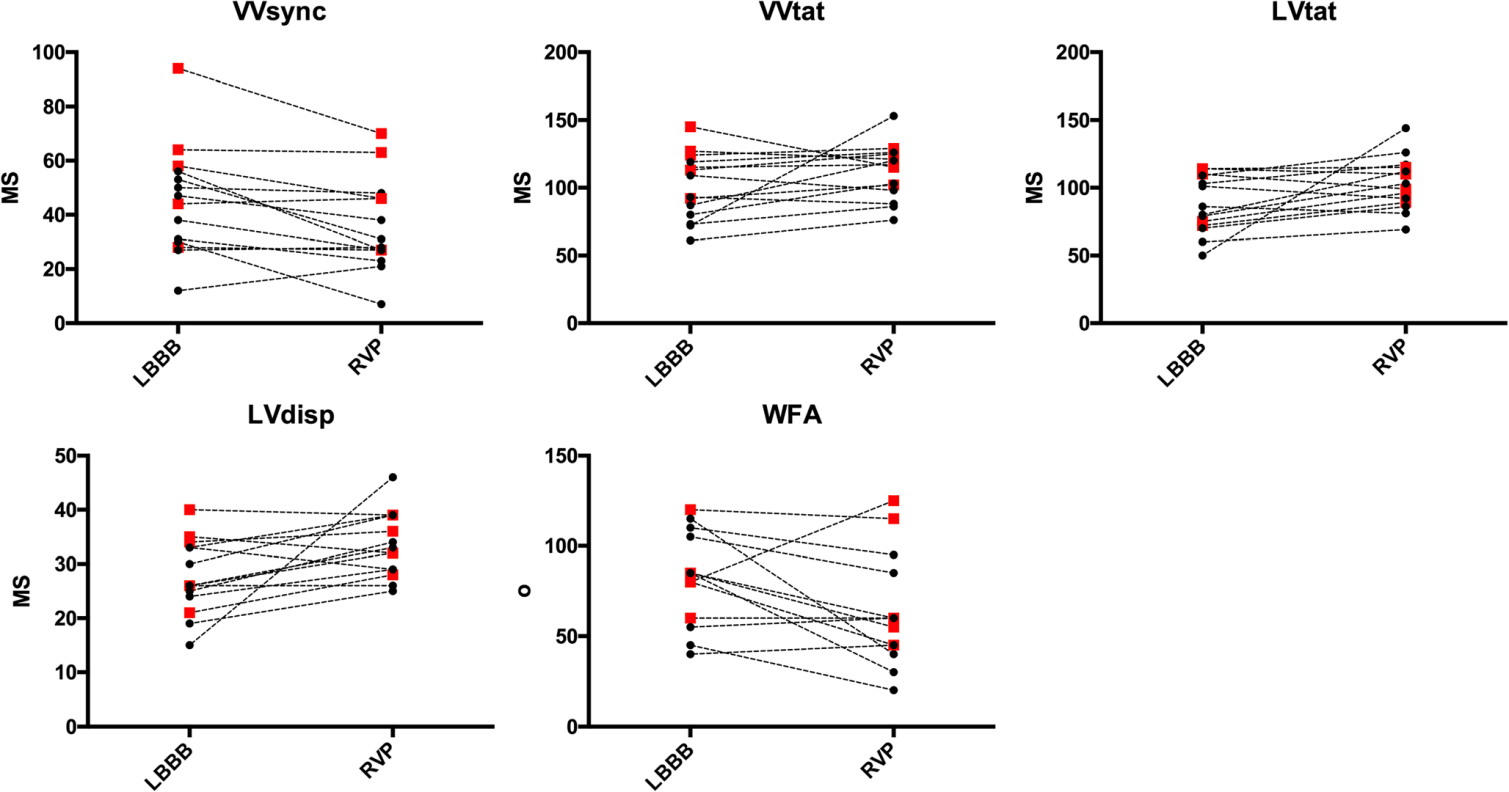
Individual changes in activation times and wave from angles. The black dots represent right ventricular apical pacing and the red squares represent right ventricular septal pacing. Abbreviations as per Fig. 2

When comparing activation time parameters between intrinsic (LBBB) and RVP, there was a significant increase in LVtat and LVdisp with RVP (87.4 ± 21.1 ms vs. 102.8 ± 19.6 ms, *p* = 0.046 and 27.6 ± 6.9 ms vs. 33.4 ± 5.9 ms, *p* = 0.025 respectively); there was no significant change in VVtat but a significant reduction in VVsync (45.1 ± 20.2 ms vs. 35.9 ± 17.1 ms, *p* = 0.01). The reduction in VVsync and increase in LVdisp were also present in the apical group analysis (38.2 ± 14.5 ms vs. 27.8 ± 11.3 ms, *p* = 0.035 and 25.7 ± 6.0 vs. 33.3 ± 6.9 ms, *p* = 0.046 respectively), with no statistical change in VVtat or LVtat. However, analysing the data for the septal pacing group demonstrated no difference between LBBB and pacing for any activation parameters. The activation wave front angle rotated anticlockwise from LBBB with RVP (82.5 ± 25.2^o^ vs. 62.1 ± 31.7^o^, *p* = 0.026) and RVAP (76.7 ± 28.9^o^ vs. 50.0 ± 27.6^o^, *p* = 0.049) with no significant change with RVSP (93.0 ± 13.5^o^ vs. 84.0 ± 28.4^o^, *p* = 0.548) (Table 2, Figs. 2 and 3).

**Table 2:** Activation times and wave front angles (WFA) for intrinsic left bundle branch block (LBBB) and right ventricular pacing (RVP). Results further broken down into right ventricular apical pacing group (apical group) and right ventricular septal pacing group (septal group)

	• All patients LBBB	RVP	*p* value	Apical group LBBB	RVP	*p* value	Septal group LBBB	RVP	*p* value
VVsync (ms)	45.14 ± 20.2	35.9 ± 17.1	0.010	38.2 ± 14.5	27.8 ± 11.3	0.035	57.6 ± 24.6	50.4 ± 16.8	0.210
VVtat (ms)	100.7 ± 24.6	111.4 ± 20.4	0.141	89.9 ± 20.6	107.4 ± 24.0	0.091	120.2 ± 8.7	118.4 ± 10.5	0.827
LVtat (ms)	87.4 ± 21.1	102.8 ± 19.6	0.046	82.0 ± 20.0	103.3 ± 23.8	0.066	97.0 ± 21.5	101.8 ± 10.6	0.478
LVdisp (ms)	27.6 ± 6.9	33.4 ± 5.9	0.025	25.7 ± 6.0	33.3 ± 6.9	0.046	31.2 ± 7.6	33.4 ± 4.2	0.319
WFA (^o^)	82.5 ± 25.2	62.1 ± 31.7	0.026	76.7 ± 28.9	50.0 ± 27.6	0.049	93.0 ± 13.5	84.0 ± 28.4	0.548

*VVsync* right/left ventricular electrical synchrony, *VVtat* biventricular total activation time, *LVtat* left ventricular total activation time, *LVdisp* left ventricular dispersion of activation, *WFA* wave front angle

**Fig. 4:**
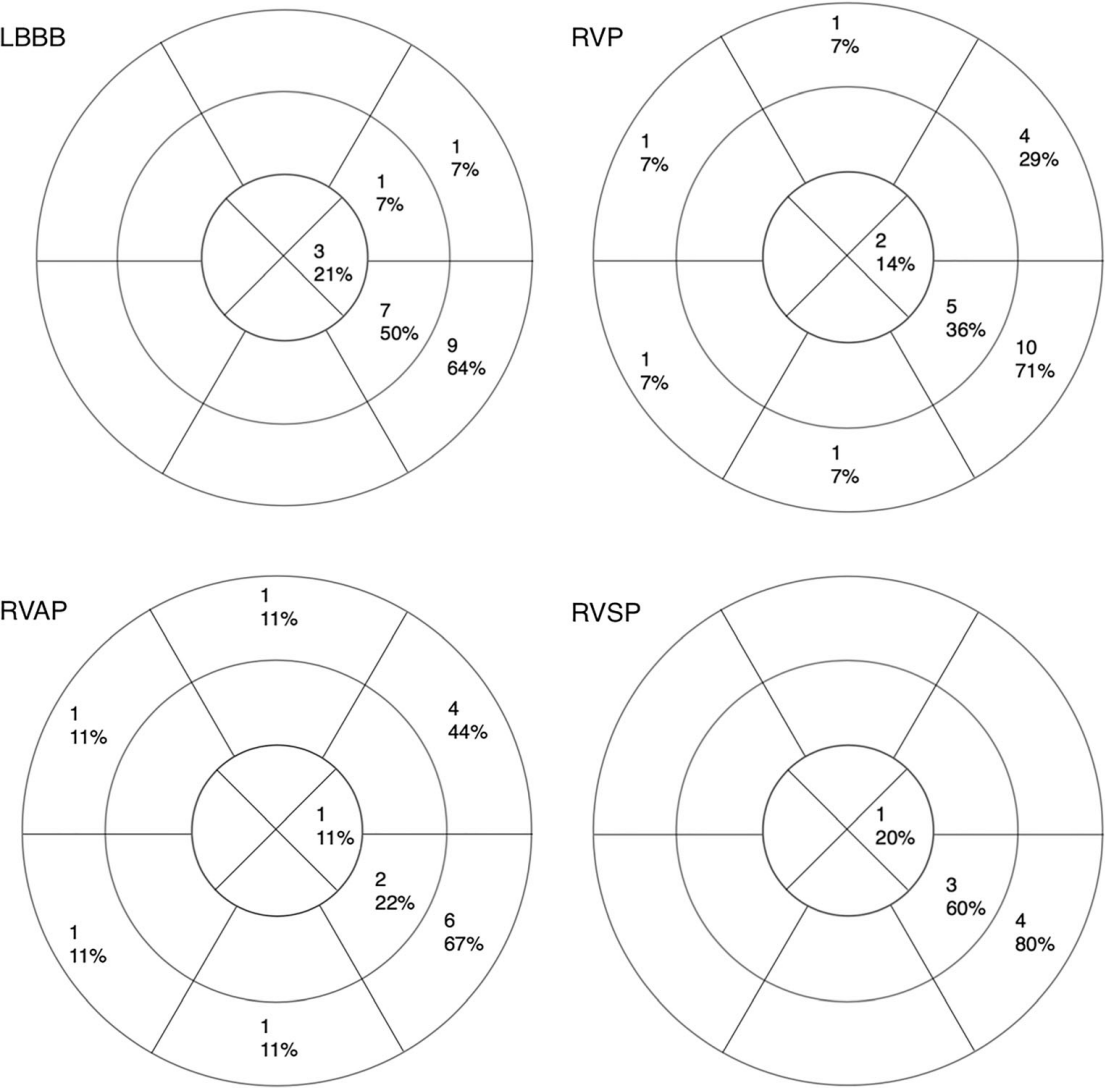
Sixteen segment American Heart Association models depicting the absolute number and percentage of patients with each segment as the final or one of the final activated segment(s) in intrinsic LBBB, right ventricular pacing (RVP), right ventricular septal pacing, and right ventricular apical pacing

The last segment or segments activated in LBBB, all RV pacing patients, RVAP, and RVSP are represented in Fig. 4. In LBBB activation, the number of AHA segments activated with the final isochrone was 1.5 ± 0.8 segments vs. 1.8 ± 1.4 segments for RVP (*p* = 0.80).

The analysis was repeated in the 8 ischemic patients in whom 5 had apical pacing and 3 had septal pacing. Baseline QRS duration was 160.5 ± 18.5 ms and ejection fraction was 25.5 ± 5.37% with intrinsic activation times of 43.1 ± 18.5 ms for VVsync, 105.1 ± 24.4 ms for VVtat, 93.3 ± 21.5 ms for LVtat, and 29.9 ± 6.6 ms for LVdisp. The baseline wave front angle was 78.8 ± 28.9^o^. RV pacing led to a significant change in LVdisp (to 33.5 ± 5.5 ms, *p* = 0.049); there were no other significant changes in these 8 patients when analysed as a whole or separated into the RVAP and RVSP groups.

### *In silico* modelling

**Fig. 5:**
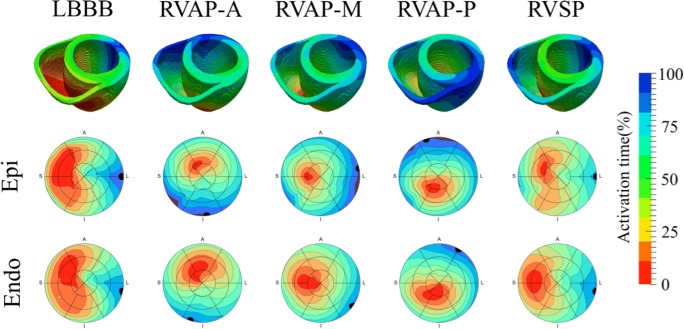
The spread of electrical activation was simulated in intrinsic LBBB, pacing at the RV apex at anterior (RVAP-A), middle (RVAP-M) and posterior (RVAP-P) sites, and pacing at the mid wall of the RV septum (RVSP). The latest activation regions (black circles) are shown on the 16 segment American Heart Association models of the activation across the LV epicardium and endocardium

The activation times were normalized with respect to the VVtat (biventricular total activation time). The Pearson correlation of the activation times across the mesh was then calculated for the intrinsic LBBB model in comparison with the three RVAP and RVSP simulations. Consistent with the observations seen in the patient studies with electroanatomical mapping in the clinic, it was found that the RVSP simulation had a higher correlation to the LBBB simulation in comparison with the RVAP simulations at the anterior, middle, and posterior sites at the RV apex (81% vs 26–73%) (Fig. 5). The latest activation region in the LV free wall did not shift significantly with RVSP (1 mm); however, RVAP caused a change in the latest activation region by 10–41 mm.

## Discussion

In this study, we have observed significant differences between activation patterns and timings between LBBB and RVP in patients studies with non-invasive imaging and with in-silico computer modelling. The main findings are as follows:

1. Biventricular total activation time was the same with RVP and LBBB, however LV total activation time (LVtat) was longer with RV pacing than LBBB. This difference was driven by those patients with RV apical pacing with no difference in LVtat between RV septal pacing and LBBB.
2. Left/right electrical synchrony was more prolonged in LBBB than in RVP; again this difference was significant with RV apical but not RV septal patients studied.
3. The dispersion of activation within the LV was greater with RV apical but not RV septal pacing compared to LBBB.
4. The direction of travel of the activation wave front was significantly different in with RV apical pacing compared to LBBB with the wave front angle rotated anticlockwise during RVP (this difference was not present in the septal pacing group.)
5. RV septal pacing led to a similar spread of terminally activated segments to LBBB, with the majority of sequences terminating in the basal and mid inferolateral segments. RV apical pacing tends to lead to the basal segments predominating as the terminal activated segments.
6. These changes were consistent with the *in silico* modelling with a higher correlation between the intrinsic LBBB model when pacing at the mid septal wall in the RV than when pacing at the mid, anterior, or posterior RVA (81% vs 73%, 26% or 26%). The latest activation region in the LV free wall did not shift significantly with RVS pacing (1 mm); however, RVA pacing caused a change in the latest activation region by 10–41 mm.

### Total activation times, wave front angles, and terminally activated segments

**Fig. 6:**
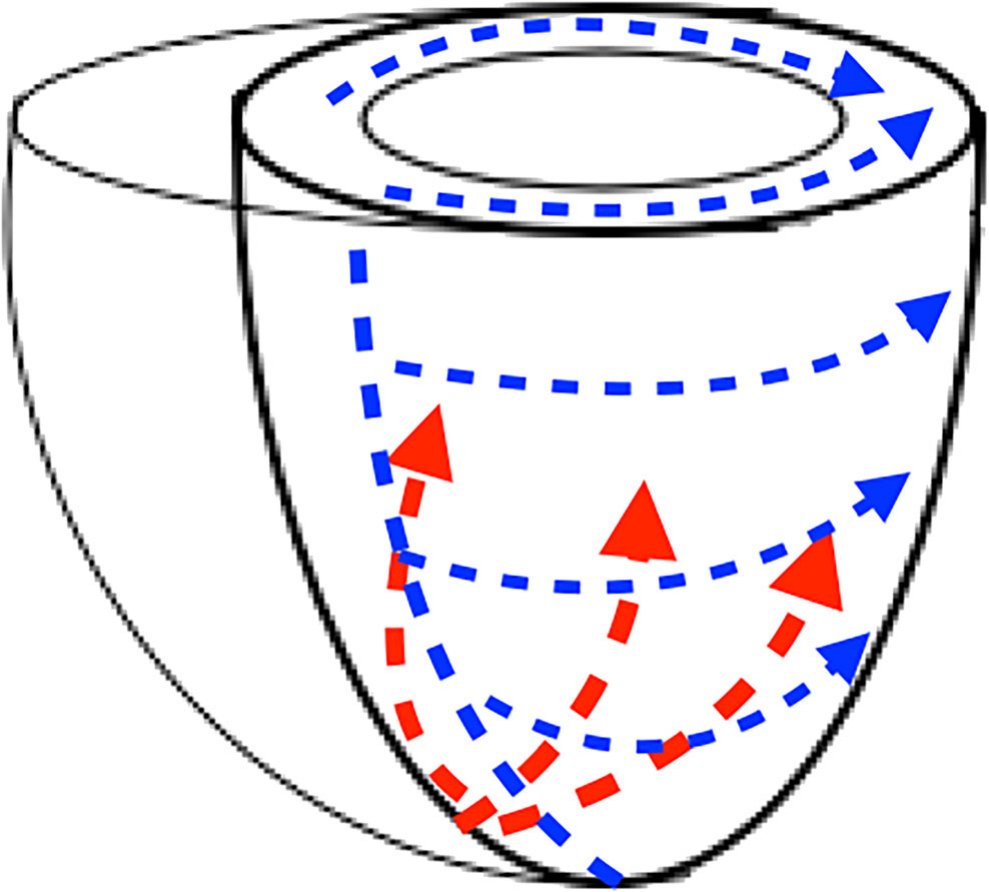
Schematic representation of the activation patterns seen in right ventricular apical pacing in red and LBBB/right ventricular septal pacing in blue

The difference in LV total activation times is intertwined with the different activation patterns between LBBB and RV apical pacing. The activation pattern in LBBB is well described with basal to apical lines of slow conduction and the lateral/inferolateral walls being the last to depolarise [17, 18]. The RV apical pacing patterns in this study are similar to those described in 2 previous body surface mapping studies in this patient group with activation propagating from apex to base with a circumferential wave front [8, 10]. In the study from Eschalier et al. [10], the difference in LVtat between apical pacing and LBBB failed to reach statistical significance (116 ± 27 vs 103 ± 22 ms, *p* = 0.06); however, the mechanism by which this difference exists can be explained simply by the greater basal to apical length than the left ventricular width; therefore, conduction takes longer from base to apex as opposed to from septal to lateral wall (Fig. 6). The anticlockwise rotation of activation wave fronts and the predominance of basal segments terminally activating with apical RV pacing are a further feature of the apical to basal pattern in RV apical pacing.

### Left/right electrical synchrony

Previous body surface mapping studies in the CRT population have termed this ventricular electrical uncoupling and demonstrated significant improved response to CRT within patients with VVsync > 50 ms [18]. The difference seen in VVsync seen between LBBB and RV apical pacing has previously been reported by Eschalier et al. [10]; however, the amount of reduction in VVsync with RV apical pacing over LBBB is significantly less in our patient group than in this previous work where VVsync was reduced from 73 ± 12 to 38 ± 21 ms (vs. 38 ± 15 ms to 28 ± 11 ms). This difference is likely due to our patients having a narrower intrinsic QRS duration than in the Eschalier study (162 ± 13 ms vs. 153 ± 21 ms). No previous comparison has been made between septal pacing and LBBB, the similarity in VVsync seen further suggests that RV septal pacing produces activation times and patterns closer to LBBB than RV apical pacing.

### Left ventricular dispersion of activation

Imaging studies with both 3D echocardiography and cardiac magnetic resonance imaging of left ventricular contraction patterns in CRT patients demonstrate improved response to CRT amongst those patients who have a greater variability or dispersion of contraction times [19, 20]. These studies show a greater systolic dyssynchrony index, measured as the standard deviation of the time to peak contraction of each segment, and leads to a more likely chance of clinical response to CRT. LVdisp measures the standard deviation of epicardial activation times within the left ventricle; as this can contain over 1000 electrograms, it is a much higher fidelity measurement of activation than these imaging measurements of contraction. This study establishes a greater dispersion of LV activation in RV apical pacing than in LBBB. The increased spread of activation times with RV apical pacing may help explain the benefit of upgrading chronic RV apical pacing patients to CRT. The lack of difference between septal pacing and LBBB LVdisp corroborates the findings of the other activation metrics suggesting that RV septal pacing can produce a very similar activation pattern to LBBB.

### *In silico* modelling

The results of the *in silico* model are in keeping with the results from patients undergoing non-invasive mapping. The model predicts that RV septal pacing will produce an activation pattern in the left ventricle which is similar to intrinsic LBBB whereas apical pacing resulted in a differing pattern of activation.

### Clinical implications

These results of the *in vivo* studies and the *in silico* modelling are important in demonstrating that with the choice of RV pacing site, the site of latest LV activation may shift significantly and this may be important in terms of left ventricular lead placement; especially if a targeted approach using electrical activation (Q-LV) is used. They suggest that operators should consider measuring Q-LV following RV lead implant rather than at baseline and perhaps target more basal LV pacing with an apical RV lead.

### Limitations

This is a small study of activation patterns in RVP and LBBB. One particular shortcoming is that there are fewer RV septal pacing patients than RV apical pacing patients. However, despite the small numbers, there is a strong signal that RV apical pacing produces a very different activation pattern to LBBB, whereas septal pacing activation leads to a similar activation to LBBB. Also, the fact that RV pacing activation pattern is directly compared with LBBB within the same patient helps to strengthen the statistical outcomes. LBBB leads to heterogeneous activation patterns [17] and ischaemic scar can particularly impact activation, again, this shortcoming is in some way overcome due to the paired analysis performed in this study. The result could also be better tested if RVAP and RVSP were assessed within the same patients; however, as our methodology required the mapping to occur after implant as opposed to intraoperatively, it was not feasible in this study. A future study assessing non-invasive activation patterns of RV apical, RV septal, and LBBB within the same patients whilst recording acute hemodynamic data would be very useful to help guide RV lead placement in CRT patients.

#### Conclusions

RV apical pacing has previously been used as a surrogate for LBBB. This study suggests that RV septal pacing may be closer to intrinsic LBBB activation than RV apical pacing. Although there is yet to be clinical data to suggest that RV lead position effects outcomes in CRT, this study suggests that the RV lead position will impact the activation pattern and therefore, attention may need to be paid to LV lead positioning on the basis of the RV pacing site. In conclusion, RV apical pacing activation differs from LBBB where RV septal pacing appears similar.

## Electronic supplementary material

ESM 1 (DOCX 19 kb)
