# Supplementary material for: Differential effect with septal and apical RV pacing on ventricular activation in patients with left bundle branch block assessed by non-invasive electrical imaging and in silico modelling
Source: J Interv Card Electrophysiol. 2019 Jun 14;57(1):115–23. doi: 10.1007/s10840-019-00567-2 (PMC7036078; doi:10.1007/s10840-019-00567-2)
Supplement: Supplementary file 1 — (DOCX 19 kb) [file 10840_2019_567_MOESM1_ESM.docx]

# Supplementary Material for Differential Effect with Septal and Apical RV Pacing on Ventricular Activation in Patients with Left Bundle Branch Block Assessed by Non-invasive Electrical Imaging and In-silico Modelling.

### Global Right/Left Ventricular Electrical Synchrony (VVsync)

*VVsync* is a statistic which indicates the degree of synchrony between the right and left ventricles. It is calculated as the mean activation time in the right ventricle minus the mean activation time in the left ventricle:

$$VVsync=\frac{\sum_{i=1}^{N_{R}} {AR}_{i}}{N_{R}}-\frac{\sum_{i=1}^{N_{L}} {AL}_{i}}{N_{L}}$$

Where:

*N_R_* is the number of points in the right ventricular region of interest

*N_L_* is the number of points in the left ventricular region of interest

*AR_i_* is an activation time value in the right ventricular region of interest

*AL_i_* is an activation time value in the left ventricular region of interest

*VVsync* is undefined if either the left or right ventricular region of interest has 0 points.

### Global BiVentricular Total Activation Time (VVtat)

VVtat is a measurement of the total time required for both ventricles to activate. It is defined as the maximum of ( mean of maximum 10% of left ventricular activation times and mean of maximum 10% of right ventricular activation times) minus the minimum of (mean of minimum 10% of left ventricular activation times and mean of minimum 10% of right ventricular activation times):

$$VVtat=MAX(\frac{\sum_{i=1}^{N_{L}} {Lmax}_{i}}{N_{L}}, \frac{\sum_{i=1}^{N_{R}} {Rmax}_{i}}{N_{R}})-MIN(\frac{\sum_{i=1}^{N_{L}} {Lmin}_{i}}{N_{L}},\frac{\sum_{i=1}^{N_{R}} {Rmin}_{i}}{N_{R}})$$

Where:

*N_L_* is 10% of the number of points in the left ventricular region of interest, rounded to the nearest integer value

*N_R_* is 10% of the number of points in the right ventricular region of interest, rounded to the nearest integer value

*Lmax_i_* is an activation time value from the maximum 10% of activations times in the left ventricular region of interest

*Rmax_i_* is an activation time value from the maximum 10% of activations times in the right ventricular region of interest

*Lmin_i_* is an activation time value from the minimum 10% of activations times in the left ventricular region of interest

*Rmin_i_* is an activation time value from the minimum 10% of activations times in the right ventricular region of interest

### Global Left Ventricular Total Activation Time (LVtat)

*LVtat* is a measurement of the total time required for all portions of the left ventricle to activate. It is defined as the average of the maximum 10% of activation times in the left ventricle minus the average of the minimum 10% of activation times:

$$LVtat=\frac{\sum_{i=1}^{N} {Amax}_{i}}{N}-\frac{\sum_{i=1}^{N} {Amin}_{i}}{N}$$

Where:

*N* is 10% of the number of points in the left ventricular region of interest, rounded to the nearest integer value

*Amax_i_* is an activation time value from the maximum 10% of activations times in the left ventricular region of interest

*Amin_i_* is an activation time value from the minimum 10% of activations times in the left ventricular region of interest

*LVtat* is undefined if the total number of samples in the left ventricular region (*N*) is less than 10. The number of points in the maximum and minimum set of activation times is always the same and is calculated by rounding to the nearest integer value equal to 10% of the total; e.g.:

| ***Total number***  ***of points*** | ***Number of maximum***  ***and minimum points*** |
| --- | --- |
| 11 – 14 | 1 |
| 15 – 24 | 2 |
| 25 – 34 | 3 |

### Global Left Ventricular Dispersion of Activation (LVdisp)

*LVdisp* is a measure of the dispersion of the activation times in the left ventricular region of interest. It is defined as the standard deviation of activation times in the left ventricular region of interest:

$$LVdisp=\sqrt{\frac{\sum_{i=1}^{N} \left( A_{i}-\bar{A} \right)^{2}}{\left( N-1 \right)}}$$

Where:

*N* is the number of points in the left ventricular region of interest

*A_i_* is an activation time value in the left ventricular region of interest

*Ᾱ* is the average activation time in the left ventricular region of interest
